# A Plant‐Based Platform for the Production of Bark Beetle Pheromones

**DOI:** 10.1111/pbi.70481

**Published:** 2025-12-17

**Authors:** Abraham Ontiveros‐Cisneros, Jule Salfeld, Sofia Paulsson, Bao‐Jian Ding, Hong‐Lei Wang, Magne Friberg, Christer Löfstedt, Olivier Van Aken

**Affiliations:** ^1^ Department of Biology Lund University Lund Sweden; ^2^ Plant Cell Biology, Faculty of Biology University of Freiburg Freiburg Germany; ^3^ Xianghu Lab Hangzhou China

**Keywords:** *cis‐*verbenol, *Dendroctonus*, *Ips*, ipsdienol, metabolic engineering, plant biotechnology, *trans‐*verbenol, α‐pinene, β‐myrcene

## Abstract

Bark beetle species of the genera *Ips* and *Dendroctonus* represent a threat to forests in both North America and Europe. Under normal circumstances, these beetles recycle dying trees into nutrients, but under certain conditions, growing populations can overcome healthy tree defenses and cause severe economic loss in forestry. The most economically relevant bark beetle species communicate with aggregation pheromones such as ipsdienol, *cis*‐verbenol and *trans‐*verbenol. These pheromones are currently used in synthetic baits as part of control strategies for bark beetles, although their chemical synthesis makes them expensive to use. Here, we explore the possibility of producing bark beetle pheromones in plant factories, since these compounds can be derived from isopentenyl diphosphate (IPP) and dimethylallyl diphosphate (DMAPP) from the mevalonic acid (MVA) and methylerythritol phosphate (MEP) pathways in plants. By the combined expression of enzymes from plants and bark beetles, we show that *Arabidopsis thaliana* can produce the intermediates β‐myrcene (ipsdienol biosynthesis pathway) and α‐pinene (verbenol biosynthesis pathway). Furthermore, we were able to produce the final products *cis‐*verbenol and *trans‐*verbenol in stably transformed Arabidopsis, without the addition of external substrates. Finally, we achieved the production of verbenone, an anti‐aggregation pheromone derived from verbenol, which deters bark beetles from a host. These results are an important step towards using plants as biofactories for a cheaper and greener production of pheromones and repellent components for artificial baits.

## 1 Introduction

Bark beetles (Coleoptera: Curculionidae: Scolytinae) under non‐outbreak circumstances have a beneficial role for the ecosystem. By consuming dead or dying trees, these beetles contribute to nutrient cycling, canopy thinning, biodiversity and soil structure. However, some bark beetle species have become forest pests causing significant economic losses (Raffa et al. 2015). In North America, *Dendroctonus ponderosae* is considered the most aggressive bark beetle, while in Europe the most detrimental species is *Ips typographus* (Fettig and Hilszczański 2015), and other species of the genera *Dendroctonus*, *Ips* and *Scolytus* may also cause severe damage. *Ips typographus* has caused 8% of all tree mortality in Europe between 1850 and 2000, causing the loss of about 124 billion m^3^ of biomass during this period (Schelhaas et al. 2003; Seidl et al. 2011). This damage is expected to increase with a growing population of bark beetles due to climate change, affecting also the carbon sequestration capacity of forests according to prediction models (Seidl et al. 2008). Contributing to bark beetle success is their symbiosis with *Ceratocystis* and *Europium* fungi, which provide nutrition and metabolic function to overcome tree defenses in return for dispersal and propagation (Raffa et al. 2015). These fungi cause blue stained wood, which translates to economic losses estimated, for instance, to be up to 400 million USD within 10 years in Japan only (Chow and Obermajer 2007; Grégoire et al. 2015).

Beetles from the genera *Dendroctonus* and *Ips* attack healthy trees and kill them through mass colonisation. At low numbers, they colonise dead or low‐vigour trees. However, large bark beetle populations can kill healthy and vigorous trees (Paine et al. 1997). Scolytinae bark beetles use aggregation pheromones to attract conspecifics to host trees to overcome tree defences, which leads to mating and resource exploitation (Fettig and Hilszczański 2015; Symonds and Gitau‐Clarke 2016). These aggregation pheromones are usually blends of chemical components that are specific to each species. For *Ips* spp., the most common components are ipsenol, ipsdienol and *cis‐*verbenol (Cognato et al. 1997; Francke et al. 1980; Giesen et al. 1984). In *Dendroctonus* spp., the main components of aggregation pheromones are frontalin, *trans‐*verbenol, *exo‐* and *endo*‐brevicomin (Renwick and Vité 1970).

Currently, control of bark beetles is accomplished in different ways, including controlled fires, removal of affected trees, placement of decoy wooden logs, insecticides in the form of sprays (in the United States), pheromone traps using aggregation pheromones as bait with the purpose of monitoring or removal of insects, and the combination of any of the above (Fettig and Hilszczański 2015). A variety of pheromone dispensers use anti‐aggregation pheromones, such as verbenone, which naturally prevent overcrowding and competition for resources (Fettig et al. 2007; Zhang and Schlyter 2004). Combining aggregation with anti‐aggregation pheromones allows a push‐pull strategy to divert beetles from high‐value tree areas (push) to traps baited with aggregation compounds (pull). An interesting alternative approach for bark beetle pest control is through predators. A study performed in China, showed successful *Dendroctonus valens* population control by releasing *Rhizophagus gradis*, a predatory beetle (Yang et al. 2014). Previous studies have shown that bark beetle pheromones not only attract conspecifics but also bark beetle predators such as *Temnochila chloridia, Enoclerus lecontei, Enoclerus sphegeus, Thanasimus formicarius and Thanasimus femoralis* (Bakke and Kvamme 1981; Boone et al. 2008; Dahlsten et al. 2003). Attracting such predators may contribute to the effectiveness of using bark beetle pheromones as a pest control strategy to prevent population outbreaks.

**FIGURE 1:**
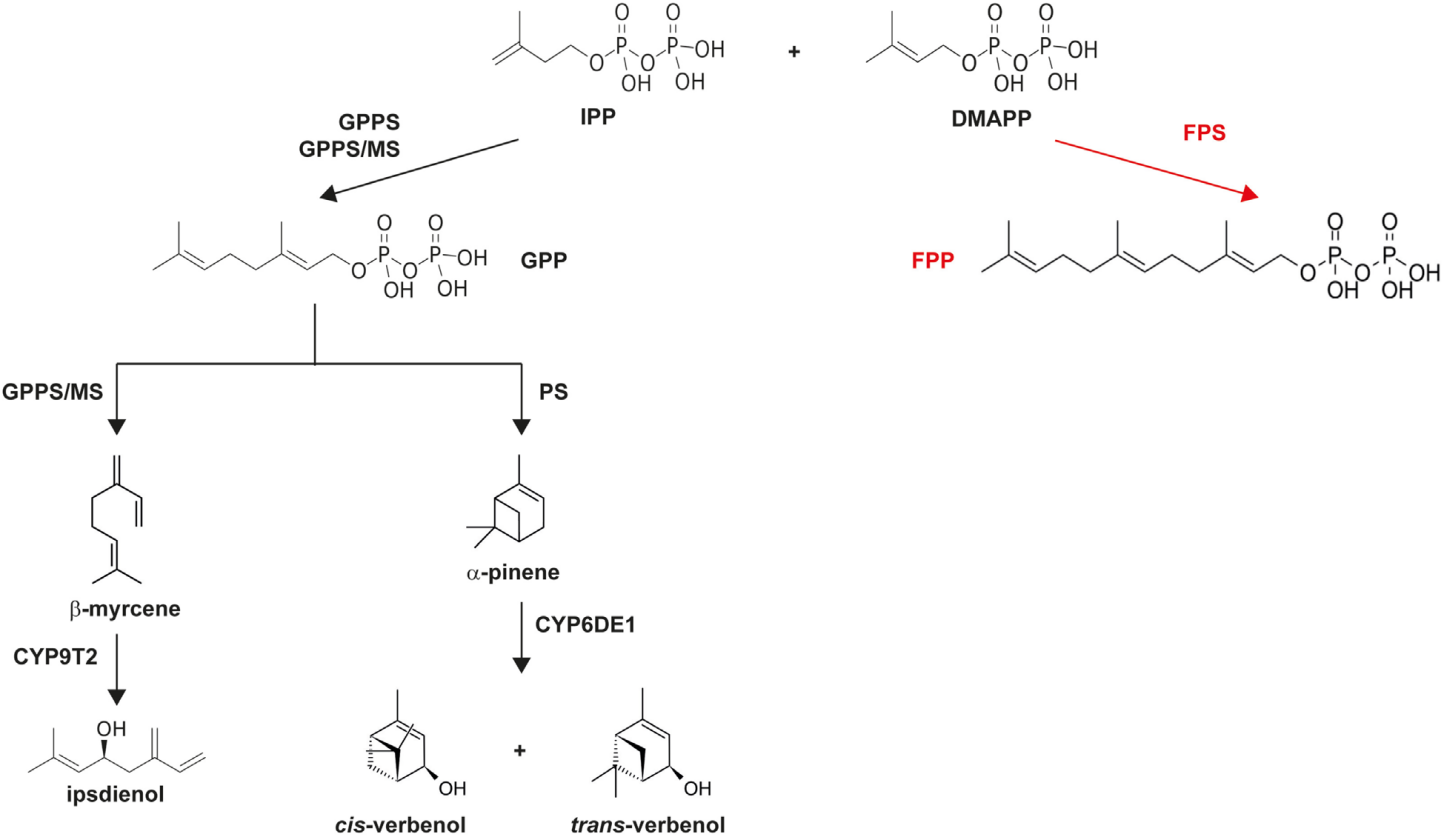
Biosynthetic pathways of ipsdienol, *cis‐*verbenol, and *trans‐*verbenol. IPP: Isopentenyl pyrophosphate, DMAPP: Dimethylallyl pyrophosphate, GPP: Geranyl diphosphate, GPPS/MS: GPP synthase‐myrcene synthase, GPPS: GPP synthase, PS: Pinene synthase, CYP9T2 & CYP6DE1: Cytochromes P450. GPP synthesis requires either GPPS or GPPS/MS. Red colour shows the competition for IPP and DMAPP substrates by Farnesyl diphosphate (FPP) synthase (FPS).

Regarding bark beetle pheromone biosynthesis, some of the most common components like ipsdienol, *cis‐*verbenol trans‐verbenol, and frontalin are derived from isopentyl diphosphate (IPP) and dimethylallyl diphosphate (DMAPP) from the mevalonic acid (MVA) pathway (Tittiger and Blomquist 2017). For ipsdienol biosynthesis (Figure 1), the bifunctional enzyme geranyl diphosphate synthase/myrcene synthase (GPPS/MS) condenses IPP and DMAPP to geranyl diphosphate (GPP) and subsequently to myrcene in *Ips pini* (Gilg et al. 2009). Finally, the cytochrome P450 CYP9T2 hydroxylates myrcene to ipsdienol (Tittiger and Blomquist 2017). To obtain enantiomeric ratios for ipsdienol to be active as a pheromone, several additional, species‐specific, steps are required, including an ipsdienol hydrogenase (Blomquist et al. 2021). In the case of *cis‐* and *trans‐*verbenol, biosynthesis of the intermediate α‐pinene (Figure 1) occurs in conifers where IPP and DMAPP from both MVA and methylerythritol phosphate (MEP) pathways can be converted to GPP by a geranyl diphosphate synthase (GPPS). Subsequently, GPP can be metabolised into α‐pinene by a pinene synthase (PS) (Phillips et al. 2003). Bark beetles can detoxify α‐pinene from the host by hydroxylating it into *cis‐* and *trans‐*verbenol by the action of the cytochrome P450 CYP6DE1 (Chiu et al. 2019). Previously it was believed that ipsdienol and verbenol were solely produced by obtaining myrcene and α‐pinene precursors from hosts, but there is evidence suggesting *de novo* biosynthesis by bark beetles themselves (Seybold et al. 2000; Seybold and Tittiger 2003; Song et al. 2013; Symonds and Gitau‐Clarke 2016). Frontalin is thought to be produced via a 20‐carbon geranylgeranyl diphosphate (GGPP) intermediate, but the full biosynthesis pathway is still unknown (Keeling et al. 2013).

As detailed before, IPP and DMAPP are precursors of some of the most dominant components of bark beetle aggregation pheromones and can be found in plants as components of the MVA (cytosolic) and MEP (plastidial) pathways (Yang et al. 2012). The aim of this study is to engineer the necessary metabolic steps to produce bark beetle aggregation pheromones in different plant species. Considering that pheromone traps are already used for bark beetle control, the generation of plants as biofactories of these compounds where pheromones can be synthesised and purified, could be an alternative to chemical synthesis, which requires catalysts such as copper, nickel and manganese and temperatures reaching 80°C (Frolova et al. 2001; Wang et al. 2006). In addition, plants could potentially be used as natural pheromone‐releasing dispensers to either attract bark beetle predators or act as lures to disperse bark beetles from high‐value woody plants. This could be implemented in tree‐based intercropping (TBI) systems with transformable pheromone‐producing plants. For example, soybean and other legumes have shown benefits to pine and other trees such as improved growth, soil nutrient content and carbon sequestration (Fan et al. 2006; Ijzerman et al. 2022; Moreira et al. 2024; Rivest et al. 2009). Both strategies seem realistic, since bark beetles prefer certain stereochemistry of the pheromones, while their predators are attracted to a wide range of enantiomeric ratios (Bakke and Kvamme 1981; Boone et al. 2008; Dahlsten et al. 2003; Symonds and Gitau‐Clarke 2016).

In this study, we used different cloning techniques to build the necessary constructs carrying the genes for the metabolic steps related to ipsdienol, *cis‐*verbenol and *trans‐*verbenol production. These constructs were transformed into *Arabidopsis thaliana* (Arabidopsis) as a proof of concept, but also *Camelina sativa* (Camelina) which is a promising platform for the biotechnological production of high‐value chemicals (Clavijo‐Bernal et al. 2024; Haslam et al. 2025; Wang et al. 2022). Apart from using wild‐type Arabidopsis, we also transformed our constructs into a *farnesyl diphosphate synthase* (*fps1*) background. Farnesyl diphosphate (FPP) synthase (FPS) catalyses the synthesis of FPP from IPP and DMAPP precursors, thus competing for substrates with the bark beetle pheromone‐producing enzymes (Figure 1). This mutant background could therefore have the advantage of decreased competition for IPP and DMAPP, the substrates of our engineered pathways. Arabidopsis contains two genes encoding an FPS: *FPS1* and *FPS2*. However, a double mutant is lethal for the plant, since FPP is an important substrate required for growth, development and defense compound production (Closa et al. 2010). We chose to use *fps1* single mutants, as FPS1 is thought to have the largest contribution to FPP production in Arabidopsis (Closa et al. 2010). In our study, as well as in the study by Closa et al. (2010), there were no apparent differences in growth or development in *fps1* compared to wild type. Plants engineered with ipsdienol, and verbenol metabolic pathways were evaluated for gene expression by qRT‐PCR and for pheromone production by GC/MS analysis. In addition, further enzymes were super‐transformed on top of the necessary metabolic steps for verbenol and ipsdienol production transformed in previous generations, with the aim of improving terpene metabolite production.

## 2 Experimental Procedures

### 2.1 DNA Assembly and Cloning

The genes introduced for verbenol synthesis were *GPPS*, *PS* and *CYP6DE1*, while ipsdienol production requires *GPPS/MS* and *CYP9T2*. The sequences for these genes were retrieved from GenBank: *GPPS* from *Picea abies* (EU432047.2), *PS* from *Pinus taeda* (AF543527.1), *CYP6DE1* from *D. ponderosae* (JQ855668.1), *GPPS/MS* (AY953508.1), and *CYP9T2* (DQ676820.1) from *I. pini* . Sequences were codon optimised for Arabidopsis and synthesised using ThermoFisher GeneArt Gene synthesis. Coding sequences *of GPPS, PS, CYP6DE1, GPPS/MS, CYP9T2* as well as a Basta resistance gene were synthesised flanked by a CaMV 35S promoter in the 5′ end, different terminators at the 3′ end and DNA recombination sequences (*attB* sites) (Figure S1). The assembly of all the required genes in a single construct was performed via MultiSite Gateway cloning (Figure S1). Plasmids used were pDONR221 as a donor vector and pXZ393b as a destination vector.

For the second round of cloning, a geranyl diphosphate synthase small subunit (*GPPS.SSU*) sequence was obtained from *Mentha × piperita* (AF182827.1), a myrcene synthase (*MyrSynt*) from *Picea abies* (AY473626.1) and a camphor oxidase (*CYP101*) from *Pseudomonas putida* (M12546.1). All these sequences were codon optimised for Arabidopsis including a 3′ end Myc‐tag and flanking *attB1/attB2* sites and synthesised by the ThermoFisher GeneArt Gene synthesis service. In the case of *CYP101*, two versions with amino acid substitutions F87W/Y96F/L244A or Y96F/L244A/V247L were created and supplemented with or without a chloroplast targeting peptide (RecA) (Cerutti et al. 1992). Individual genes were cloned via Gateway cloning using pDONR221 and pH2GW7, a destination vector conferring hygromycin resistance. To create constructs carrying two genes, *GPPS.SSU* was cloned with either *MyrSynt* or *CYP101* via Gibson Assembly and MultiSite Gateway cloning (Figure S2). Since the pH2GW7 destination vector has a 35S promoter and 35S terminator flanking the recombination sites, an RBCS terminator was added to *GPPS.SSU* and a 35S promoter to both *MyrSynt* and *CYP101* via Gibson Assembly. Later, these constructs were cloned into the pH2GW7 destination vector using MultiSite Gateway cloning (Figure S2). Before cloning, both individual and double gene constructs were amplified with primers containing *attB1* and *attB2*. For these procedures, the Gibson Assembly Cloning Kit from NEB was used, and the protocol was followed as indicated by the manufacturer. In the case of MultiSite Gateway cloning, procedures were performed according to the user manual for MultiSite Gateway Pro by Invitrogen.

All vectors were transformed via the heat‐shock method and propagated in *E. coli* DH5α on LB plates with their respective antibiotics: 50 mg/L kanamycin for pDONR221 and 50 mg/L spectinomycin for pB2GW7, pH2GW7 and pXZ393b. Subsequently, plasmids were introduced into chemically competent *Agrobacterium tumefaciens* strain GV3101 by heat‐shock transformation. Selection of transformed bacteria was done on LB plates containing 100 mg/L spectinomycin, 100 mg/L rifampicin and 40 mg/L gentamycin. The presence of the desired constructs was confirmed by full‐plasmid sequencing.

### 2.2 Stable Transformation and Selection in *A. thaliana*

*Arabidopsis thaliana* Columbia‐0 (Col‐0) and *fps1* (SALK_073576) were transformed to generate stable transgenic plant lines via floral dipping using *Agrobacterium tumefaciens* carrying the appropriate vectors as described in previous literature (Clough and Bent 1998). For this purpose, plants were grown under long day conditions (16 h light/8 h dark, approximately 120 μmol photons m^−2^ s^−1^). After transformation and senescence, resulting seeds were collected and sterilised overnight in a desiccator by using chlorine gas sterilisation with a solution of 40 mL Milli‐Q water, 6.25 mL bleach and 1.75 mL HCl. For positive transformant selection, seeds were plated on MS media at pH 5.7 with Basta 5 mg/L for pB2GW7 and pXZ393b transformed plants or with hygromycin 15 mg/L for pH2GW7 transformed plants. After 2 days of stratification at 4°C, plants were grown for approximately 10 days and survivors were transplanted to soil pots and grown under long day conditions (16 h light/8 h dark, approximately 120 μmol photons m^−2^ s^−1^).

### 2.3 Stable Transformation and Selection in *C. sativa*

In this study, *Camelina sativa* cv. Suneson was used for generation of stable transgenic plant lines via floral dipping using *A. tumefaciens* carrying the appropriate vectors as described in previous literature (Lu and Kang 2008). For this purpose, 5 seeds were sown per 10 cm pot with draining holes filled with soil mix with vermiculite and perlite at a 4:1:1 ratio. Three plants per pot were retained, discarding extra seedlings, and Camelina trays were placed in the greenhouse where the growth conditions were 16 h light/8 h dark, 400–600 μmol photons m^−2^ s‐^1^, 20/16°C (day/night) and humidity maintained at 60%. From these transformed plants, seeds were collected, densely grown on soil trays, left for 2 days of stratification at 4°C and sprayed with 100 mg/L Basta as reported in literature (Nguyen et al. 2013; Ontiveros‐Cisneros et al. 2022). Spraying started when plants reached the two‐leaf stage (approx. 1 week old), followed by two more rounds of spraying approx. every 3 days. Survivors were used for further analyses. From T2 onwards, 50 seeds per line were gas sterilised by using a chlorine gas sterilisation method with a solution of 40 mL Milli‐Q water, 6.25 mL bleach and 1.75 mL HCl and left overnight inside a desiccator. Seeds were then plated on MS media at pH 5.7 with Basta 15 mg/L or hygromycin 22.5 mg/L and homozygous lines were obtained by counting the survival rate.

### 2.4 Transient Expression in *N. Benthamiana*

*Nicotiana benthamiana* (WT) plants were used as a fast way to evaluate the construct functionality via transient expression, infiltrating the abaxial side of the leaves with *A. tumefaciens* carrying the desired vector. This procedure was performed as described by Wood et al. (2009) with 6‐week‐old plants grown under long day conditions (16 h light/8 h dark, approximately 400 μmol photons m^−2^ s^−1^) (Wood et al. 2009). Along with the desired vectors, a strain of *A. tumefaciens* carrying a *P19* gene was co‐infiltrated in every leaf, as literature shows it prevents post‐transcriptional gene silencing (Voinnet et al. 2003).

### 2.5 Gene Expression Analysis

RNA was extracted from leaf samples (~30 mg) from positive transformants with Spectrum Plant Total RNA Kit from Sigma. RNA samples were normalised to 100 ng/mL and cDNA was synthesised using Bio‐Rad iScript Reverse Transcription (RT) Kit and 500 ng of RNA per sample. RT program was as follows: 5 min at 25°C, 25 min at 42°C and 1 min at 95°C. Samples were diluted 7:1 with Milli‐Q water. For qRT‐PCR, primer pairs were designed to amplify ~120 bp products (Table S1). For Arabidopsis, Ubiquitin‐conjugating enzyme 21 (UBC) (NC_003076.8) was used as a housekeeping gene in relative expression analysis, while for Camelina *ACTIN7* (ACT7) (XM_010424636.2) was used. The qRT‐PCR mix was made with 2.5 μL Sso Advanced Universal SYBR Green Supermix, 0.25 μL primers (10 mM) and 0.25 μL Milli‐Q water, along with 2 μL of diluted cDNA. The qRT‐PCR program was performed in a CFX384 Touch Real‐Time System following previously described methods (Broda and Van Aken 2018). For all relative expression analyses, the values were normalised to untransformed wild type as reference.

### 2.6 Analysis of Volatiles—SPME and GC/MS

Single leaves (~20 mg) were taken from positive transformants and sealed in glass beakers covered with heated oven plastic (Toppits). Samples were incubated for 30 min and then the plastic cover was pierced with a Solid Phase Microextraction (SPME) Fibre SUPELCO Portable Field Sampler containing a PDMS fibre. The fibre was exposed to the leaf headspace for 30 min and retracted into the SPME sampler afterwards for overnight storage at 4°C. Volatiles were analysed using an Agilent 8890 GC System coupled with an Agilent 5977B mass selective detector. The GC system was equipped with a polar column (HP‐INNOWax, 30 m *×* 0.25 mm, 0.25 μm film thickness) and was operated using helium as a carrier gas (1 mL/min flow). The method started with the oven temperature set at 50°C for 3 min, then a temperature ramp of 10°C/min until reaching 230°C and holding it for 1 min. Standard compounds were acquired from Sigma Aldrich except for *trans‐*verbenol, ipsdienol and verbenone, which were available from Lund University's Pheromone Group compound library at the Biology Department. Detection of pathway intermediates and end‐products was performed by comparing their retention times to chemically produced standards exposed to the same GC/MS conditions and comparing the corresponding mass spectra to both standard compound and the National Institute of Standards and Technology (NIST17) database library (Figure S4). Software used for total ion chromatogram (TIC) and mass spectra analysis was Enhanced ChemStation MSD ChemStation F.01.03.2357 from Agilent Technologies Inc. The target compound peaks were shown in the extracted ion chromatograms (Figure S4B). The same software was used for the integration of peak areas to estimate volatile emissions from the plant tissue. This estimation was based on the known amount of standard compound injected into the GC device.

## 3 Results

### 3.1 Expression of Verbenol‐ and Ipsdienol Pathway Enzymes Results in the Production of Intermediates α‐Pinene and β‐Myrcene, and Final Product Verbenol

**FIGURE 2:**
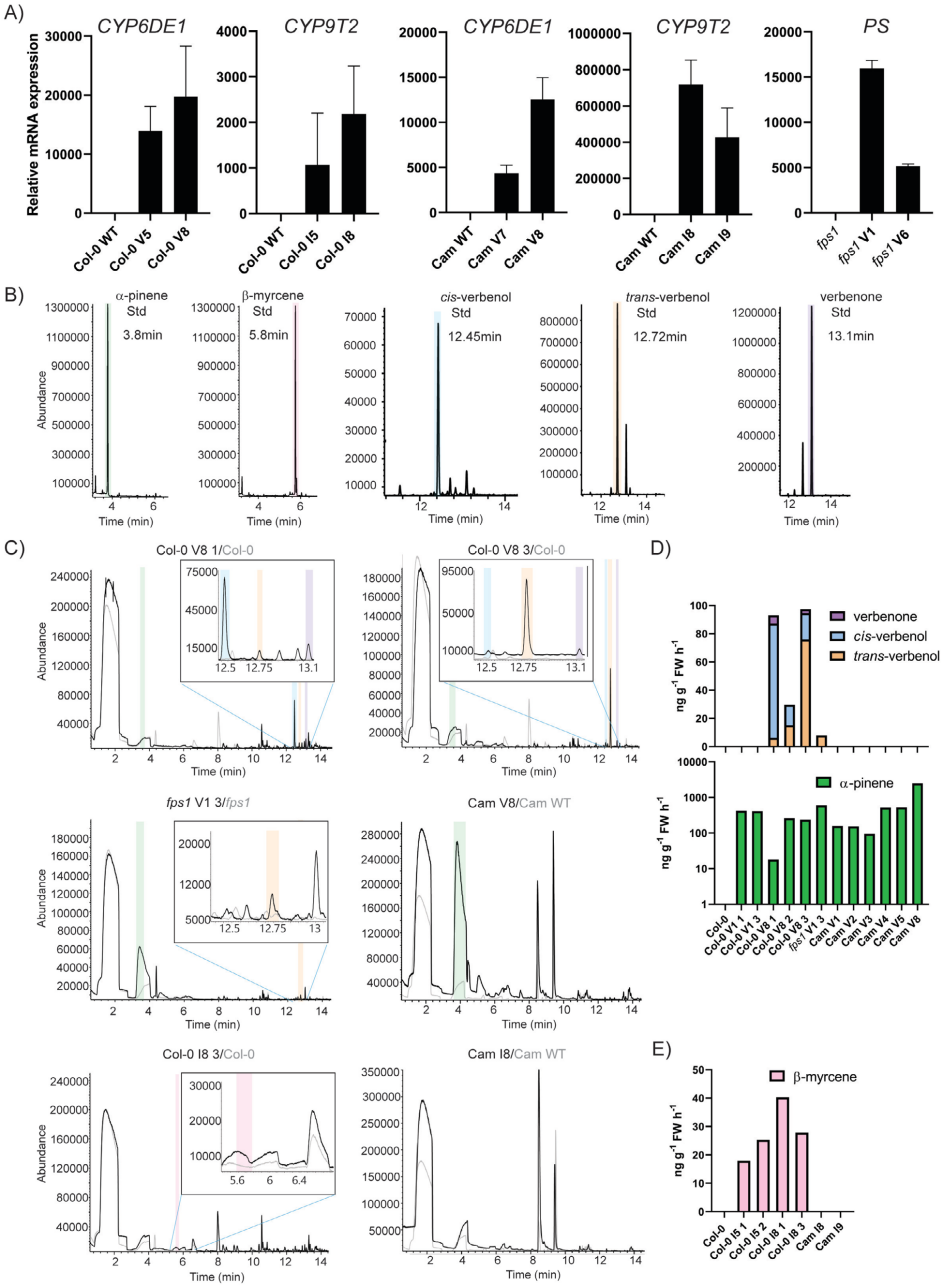
Transcription analysis and volatile profiling of lines carrying Verbenol (V) and Ipsdienol (I) pathways constructs. (A) Relative mRNA expression of verbenol (*CYP6DE1/PS*) and ipsdienol (*CYP9T2*) pathway genes in T1 Arabidopsis (Col‐0), *fps1* Arabidopsis *(fps1)* and Camelina (Cam) backgrounds. WT untransformed plants are used as reference to normalise mRNA expression. (B) α‐pinene (intermediate compound in the verbenol pathway), β‐myrcene (intermediate compound in the ipsdienol pathway), *cis*‐verbenol, *trans*‐verbenol and verbenone (verbenol derivative) standard compound GC/MS retention time. (C) Gas chromatograms of verbenol pathway construct (V)‐transformed Col‐0, *fps1* and Camelina (Cam); and ipsdienol pathway construct (I)‐transformed Col‐0 and Camelina, Coloured shading indicates detection of specific compounds at their expected retention time as follows: Green: α‐pinene at 3.80 min; pink β‐myrcene at 5.8 min; blue: *Cis*‐verbenol at 12.45 min; orange *trans*‐verbenol at 12.72 min; purple: Verbenone at 13.10 min. Overlayed chromatograms in grey represent a corresponding untransformed wild type (WT). (D, E) Estimated emission of bark beetle pheromones and intermediates in verbenol (D) and ipsdienol (E) pathway‐transformed Col‐0, *fps1* and Camelina individuals in nanogram per gram of fresh tissue (FW) per hour.

Terpenoid production in plants starts from IPP and DMAPP as substrates from the MVA and MEP pathways. For verbenol production, codon‐optimised *GPPS*, *PS* and *CYP6DE1* were cloned into a single plasmid vector and transformed into Arabidopsis Col‐0, *fps1* and Camelina backgrounds. Ipsdienol pathway metabolic engineering involved the integration of codon‐optimised *GPPS/MS* and *CYP9T2* into a plasmid vector and subsequent transformation into the above‐listed plant backgrounds. The lines with the highest mRNA abundance for each plant background and each pathway were selected for further analysis (Figure 2A). In the case of *fps1*, positive transformants were only obtained for the verbenol pathway construct.

Volatile production in transformed plants was evaluated with SPME and gas chromatography/mass spectrometry (GC/MS). The retention times of standard compounds of the pathway intermediates, final products and verbenone, a derivative of verbenol, were determined using the same method as for plant individuals (Figure 2B). In the chromatograms of untransformed Col‐0 and Camelina (Figure 2C), a wide peak was observed around the retention time of α‐pinene. Searching the spectrum against the NIST17 database indicated this peak was silanol, a common component of gas chromatography columns. No products or intermediate steps related to ipsdienol or verbenol production were detected in these untransformed controls. In ipsdienol pathway‐transformed Col‐0, β‐myrcene was detected (*N* = 4) at the expected retention time and its emission estimated to be up to 40.3 ng g^−1^ fresh weight (FW) h^−1^ from integration analysis for one of the individuals (Figure 2E, Table S4). However, none of the plants that produced myrcene showed detectable amounts of ipsdienol. These results show that the dual‐functional enzyme GPPS/MS from *I. pini* can produce β‐myrcene also in plant tissues. However, the final product ipsdienol could not be detected in any of the transgenic lines, not even in those with detectable amounts of β‐myrcene. This suggests that either CYP9T2 from *I. pini* cannot efficiently produce ipsdienol in plant cells, or that β‐myrcene availability is insufficient to drive ipsdienol production by *CYP9T2*. Alternatively, any produced ipsdienol may be rapidly degraded or converted into other compounds.

In the case of verbenol pathway‐transformed Col‐0, the α‐pinene intermediate was detected at the expected retention time (Figure 2C,D). Despite the silanol background peak at an overlapping retention time, α‐pinene could be observed visually due to the altered peak shape, which was less skewed to the right in the presence of α‐pinene (comparison to wild type Figure 2C). The presence of α‐pinene was further confirmed by subtracting background silanol ions from the mass spectra (Figure S4A). Final products *trans*‐verbenol (*N* = 3) and *cis*‐verbenol (*N* = 3) and their derivative verbenone (*N* = 2) were also detected at their expected retention times for several individuals (Figure 2D, Table S4). Interestingly, although these individuals originated from the same parental line and leaves were harvested at a similar developmental stage, differences in emission composition could be observed. For instance, the individual with highest *cis*‐verbenol emission (80.9 ng g^−1^ FW h^−1^) showed the lowest emission for the precursor α‐pinene (18.0 ng g^−1^ FW h^−1^), while another individual showed an emission of 75.9 ng g^−1^ FW h^−1^ *trans*‐verbenol with a substantial α‐pinene emission (239.3 ng g^−1^ FW h^−1^). The individual with highest α‐pinene emission (260.6 ng g^−1^ FW h^−1^) however showed a low emission of both verbenol isoforms (14–15 ng g^−1^ FW h^−1^). Some verbenol pathway‐transformed Col‐0 individuals also produced the anti‐aggregation pheromone verbenone, in particular the lines that showed the highest production of verbenol (Figure 2D, Table S4). This is likely due to auto‐oxidation of verbenol to verbenone, as no specific enzyme that catalyses this conversion is known (Frühbrodt et al. 2024). For verbenol pathway‐transformed *fps1*, α‐pinene (*N* = 1) and *trans*‐verbenol (*N* = 1) were detected at the expected retention time. For the individual *fps1* V1 3, volatile emission was estimated at 596.7 ng g^−1^ FW h^−1^ for α‐pinene and 8.0 ng g^−1^ FW h^−1^ for *trans*‐verbenol (Figure 2D, Table S4). In Camelina, ipsdienol pathway‐transformed plants did not emit detectable amounts of intermediate β‐myrcene nor ipsdienol, despite the high mRNA relative expression levels, while verbenol pathway‐transformed Camelina emitted α‐pinene (*N* = 6). Individual Cam V8 emitted 2.50 μg g^−1^ FW h^−1^, the highest amount measured in this study (Figure 2D). Surprisingly, no verbenol emission could be detected in Camelina.

Additionally, transient transformation of *N. benthamiana* leaves was performed with the verbenol pathway construct and we analysed the samples by qRT‐PCR and GC/MS. Similarly to the results obtained from stably transformed Camelina, we detected α‐pinene (468.1 ng g^−1^ FW h^−1^) but no verbenol (Figure S3).

These results show that CYP6DE1 from *D. ponderosae* can produce both *cis*‐ and *trans*‐verbenol in the Arabidopsis background. Verbenone, a derivative of these final products was also observed in the volatile emission profile of the plants with the highest verbenol emission, suggesting a subsequent oxidation of these pheromones. In some of the individuals, only α‐pinene could be detected, even when *CYP6DE1* was highly expressed at the mRNA level. This might indicate that certain conditions may need to be met in the plants' environment or development for the proper production of the final compounds.

### 3.2 *GPPS.SSU* Does Not Consistently Improve α‐Pinene/Verbenol Production, While *CYP101* Expression Favours *Trans*‐Verbenol Production

In some plant species, GPPS cooperates with a GPPS small subunit (GPPS.SSU) (Yin et al. 2017) that is missing in, for example, Arabidopsis. Co‐expression of GPPS.SSU with GPPS in transgenic lines was reported to significantly boost GPP availability for subsequent metabolic enzymes (Yin et al. 2017). As both ipsdienol and verbenol are produced from GPP, we aimed to introduce GPPS.SSU from *Mentha × piperita* into the highest expressing (at mRNA level) ipsdienol pathway and verbenol pathway transgenic individuals.

As not all the individuals transformed with the verbenol pathway construct, carrying *CYP6DE1* from *D. ponderosae*, produce verbenol in a consistent fashion, we looked for alternative enzymes that could convert α‐pinene into verbenol more efficiently. Interestingly, a previous study reported that a modified camphor oxidase CYP101 from the bacterium *Pseudomonas putida* was able to use α‐pinene as a substrate and produce verbenol in vitro (Bell et al. 2003). Two variants were reported with amino acid changes F87W/Y96F/L244A (CYP101 FW) and Y96F/L244A/V247L (CYP101 YF), which produced variable amounts of *cis‐* and *trans‐*verbenol (Bell et al. 2003).

**FIGURE 3:**
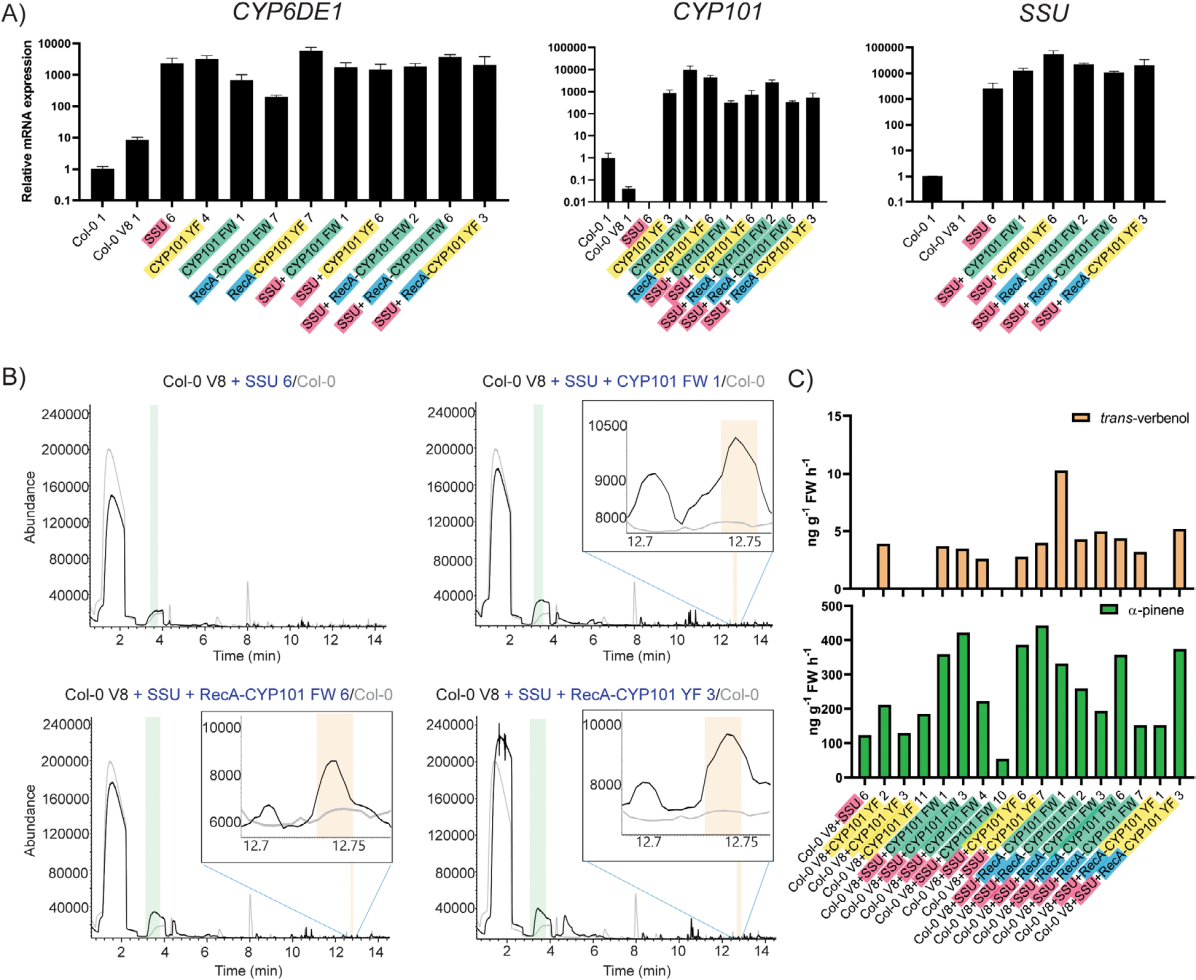
Transcription analysis and volatile profiling of 2nd order transgenic Col‐0 V8 plants transformed with GPPS.SSU (SSU) and CYP101. (A) Relative mRNA expression of a verbenol (*CYP6DE1*) pathway gene, *CYP101* and *SSU* in T1 2nd order transgenic Col‐0 V8. WT untransformed plants are used as reference to normalise mRNA expression. (B) Representative gas chromatograms of 2nd order transgenic Col‐0 V8 individuals transformed with different variants of *CYP101* and *GPPS.SSU*. SSU: GPPS.SSU, V: Verbenol pathway construct. FW: CYP101 F87W/Y96F/L244A and YF: CYP101 Y96F/L244A/V247L. Coloured shading indicates detection of specific compounds at their expected retention time: Green: α‐pinene at 3.80 min; orange: *Trans‐*verbenol at 12.72 min. Overlayed chromatograms in grey represent untransformed Col‐0. (C) Estimated emission of bark beetle pheromones and intermediates in 2nd order transgenic Col‐0 V8 individuals in nanogram per gram of fresh tissue (FW) per hour.

**FIGURE 4:**
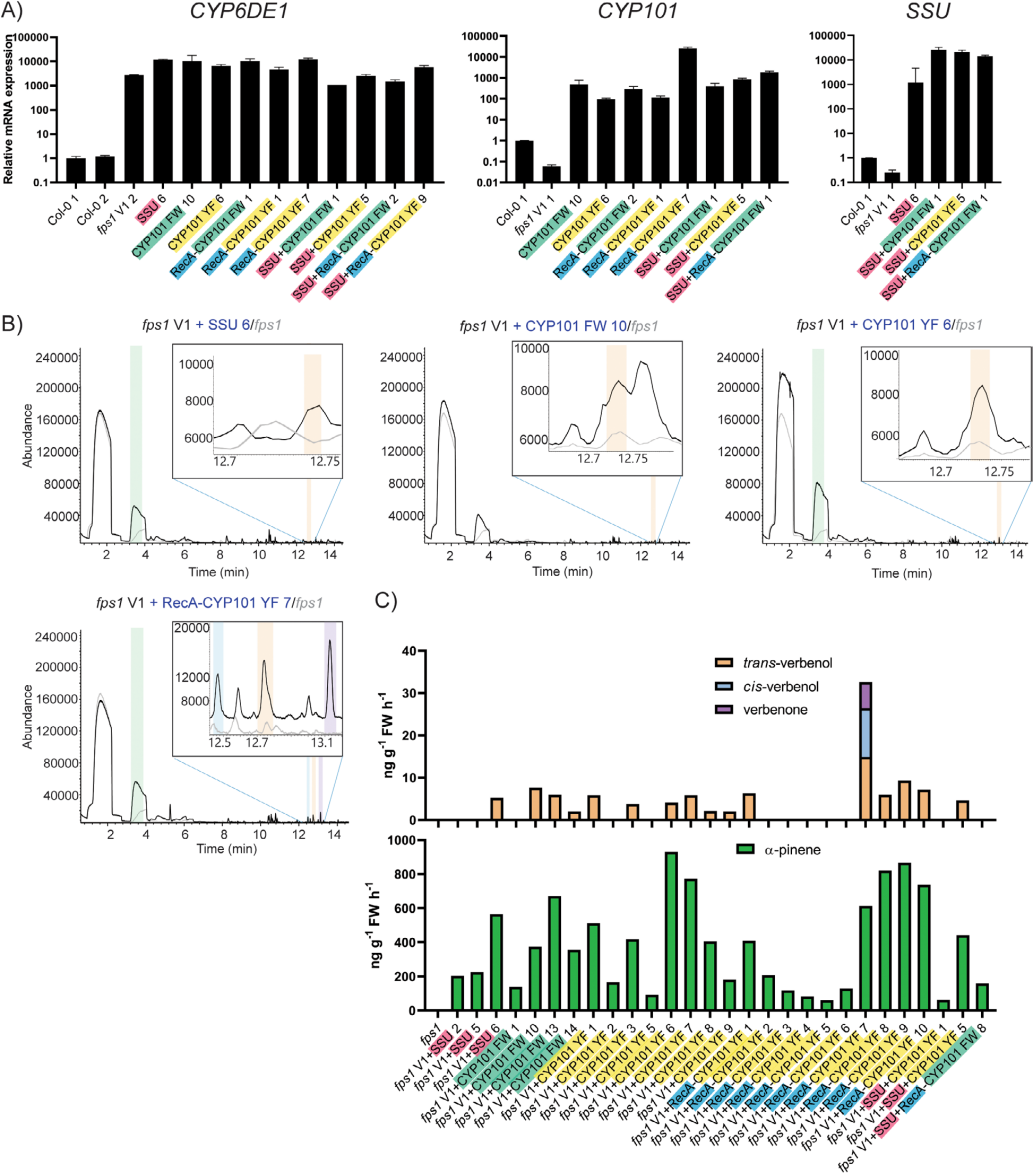
Transcription analysis and volatile profiling of 2nd order transgenic *fps1* V1 plants transformed with GPPS.SSU (SSU) and CYP101. (A) Relative mRNA expression of a verbenol (*CYP6DE1*) pathway gene, *CYP101* and *SSU* in T1 2nd order transgenic *fps1* V1. WT untransformed plants are used as reference to normalise mRNA expression. (B) Representative gas chromatograms of 2nd order transgenic *fps1* V1 individuals transformed with different variants of *CYP101* and *GPPS.SSU*. SSU: GPPS.SSU, V: Verbenol pathway construct. FW: CYP101 F87W/Y96F/L244A and YF: CYP101 Y96F/L244A/V247L. Coloured shading indicates detection of specific compounds at their expected retention time: Green: α‐pinene at 3.80 min; blue: *Cis*‐verbenol at 12.45 min; orange: *Trans*‐verbenol at 12.72 min; purple: Verbenone at 13.10 min. Overlayed chromatograms in grey represent untransformed *fps1*. (C) Estimated emission of bark beetle pheromones and intermediates in 2nd order transgenic *fps1* V1 individuals in nanogram per gram of fresh tissue (FW) per hour.

**FIGURE 5:**
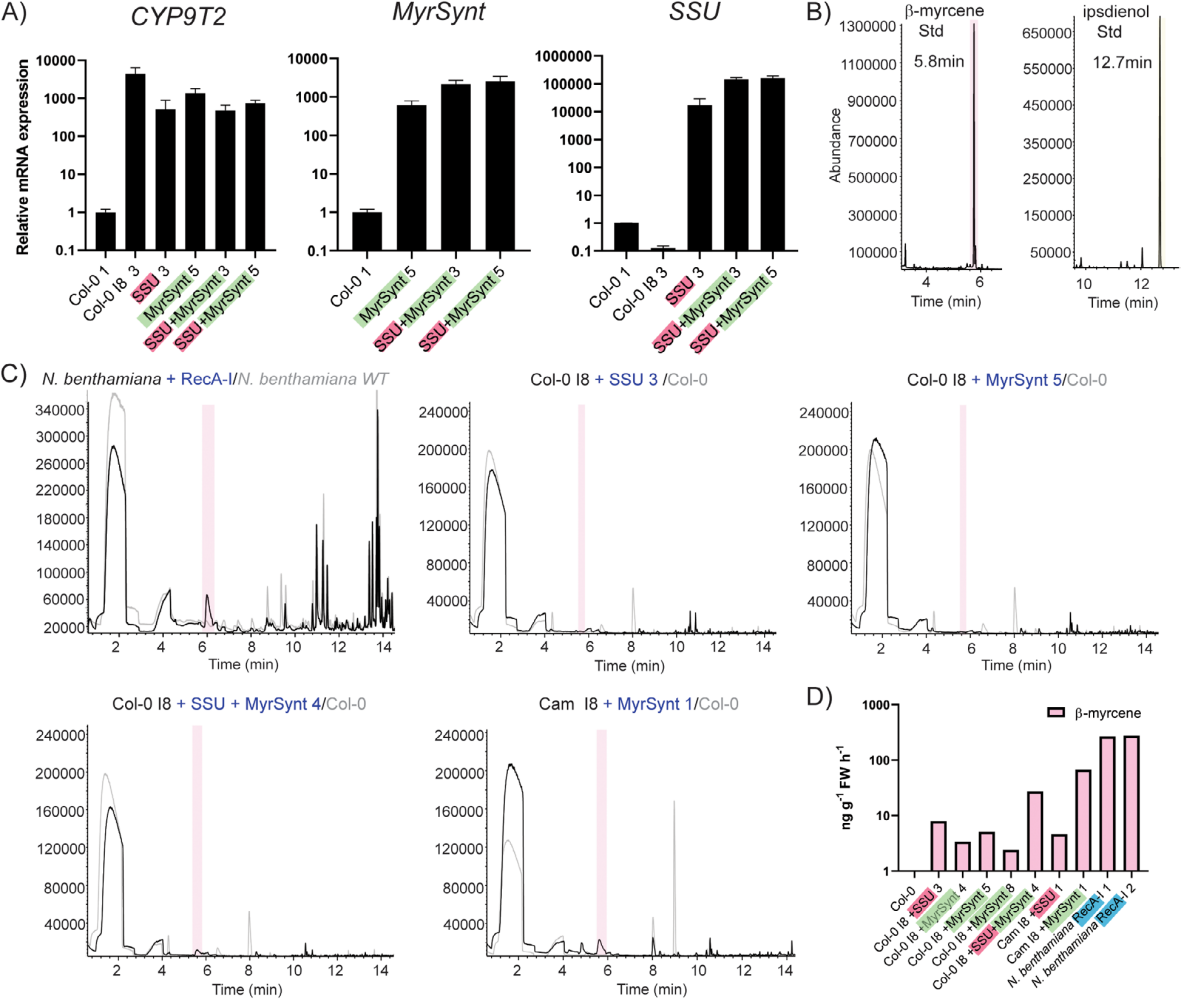
Transcription analysis and volatile profiling of 2nd order transgenic Col‐0 and Camelina (Cam) I8 plants transformed with GPPS.SSU (SSU) and *P. abies* myrcene synthase (MyrSynt), and *N. benthamiana* infiltrated with Rec‐I. (A) Relative mRNA expression of an ipsdienol (*CYP9T2*) pathway gene, *MyrSynt* and *SSU* in T1 2nd order transgenic Col‐0 I8. WT untransformed plants are used as reference to normalise mRNA expression. (B) β‐myrcene (intermediate compound in the ipsdienol pathway), and ipsdienol standards GC/MS retention time. (C) Gas chromatogram of *N. benthamiana* infiltrated with ipsdienol pathway genes targeted to the chloroplast (RecA‐I) and gas chromatograms of 2nd order transgenic Col‐0 I8 individuals transformed with *MyrSynt* and *SSU*. SSU: GPPS.SSU, MyrSynt: Myrcene synthase, I: Ipsdienol pathway construct. Coloured shading indicates detection of specific compounds at their expected retention time: Pink: β‐myrcene at 5.80 min; yellow: Ipsdienol at 12.72 min. Overlayed chromatograms in grey represent untransformed Col‐0. (D) Estimated emission of bark beetle pheromones and intermediates in 2nd order transgenic Col‐0 I8 individuals in nanogram per gram of fresh tissue (FW) per hour.

For the verbenol pathway, the best lines in terms of mRNA relative expression and metabolite detection were Col‐0 V8 and *fps1* V1 (Figure 2A,D, Table S4). For the ipsdienol pathway, the best performing line was Col‐0 I8 (Figure 2A,D). These lines were used for super‐transformation with the additional genes to improve ipsdienol and verbenol production. To increase the availability of the pathway intermediates α‐pinene and β‐myrcene, we introduced GPPS.SSU into the mentioned ipsdienol and verbenol pathway‐transformed lines. To test the activity of the modified camphor synthase CYP101 for verbenol production in plants, we transformed *CYP101 FW* or *CYP101 YF*, with or without *GPPS.SSU* and with or without the chloroplast targeting sequence RecA, into Col‐0 V8 and *fps1* V1. These 2nd order transgenic (offspring from the *GPPS.SSU* and *CYP101* super‐transformed Col‐0 V8, *fps1* V1 and Col‐0 I8 lines) T1 plants were selected under hygromycin antibiotic and transgene expression was confirmed via qRT‐PCR analysis. For the 2nd order transgenic Col‐0 V8 and *fps1* V1 lines, *CYP6DE1* was used as a reference to evaluate the presence of the original verbenol pathway construct, while *CYP101* variants and *GPPS.SSU* are the two newly transformed genes. Multiple lines, with high relative mRNA expression, were selected for volatile analysis using SPME and GC/MS (Figures 3, 4, 5).

Evaluation of the 2nd order transgenic plants showed that most individuals expressed *GPPS.SSU* (*N* = 16) and/or *CYP101* (*N* = 22) variants as expected (Figure 3A). First, we considered the effect of *GPPS.SSU* expression on α‐pinene emission in Col‐0. Seventeen individuals displayed production of α‐pinene, although not above the emissions of non‐super‐transformed individuals (compare Figure 2D vs. Figure 3C). In the *GPPS.SSU*‐expressing Col‐0 lines *trans‐*verbenol was commonly detected (*N* = 9), though at lower emission rates (approx. 3–5 ng g^−1^ FW h^−1^), while *cis‐*verbenol or verbenone was never detected in Col‐0 (Figure 3C).

For the individuals with CYP101 YF, three out of 11 showed α‐pinene emission and only one showed *trans*‐verbenol. None of the positive transformants carrying the CYP101 FW variant alone showed terpene emission (Table S4). As for the combination of SSU + CYP101 FW, four out of 10 plants showed α‐pinene emission and three showed *trans*‐verbenol. Two out of 14 plants with the combination SSU + CYP101 YF showed emission of both α‐pinene and *trans*‐verbenol. Regarding individuals expressing only chloroplast‐targeted *CYP101* (*RecA‐CYP101*), no plants showed emission of α‐pinene nor verbenol. However, when combined with SSU, *RecA‐CYP101*‐expressing plants showed emission of α‐pinene and *trans*‐verbenol. Five out of 10 individuals emitted α‐pinene and *trans*‐verbenol for the SSU + RecA‐CYP101 FW combination (Table S4). For the SSU + RecA‐CYP101 YF combination two plants out of seven emitted α‐pinene, while only one emitted *trans*‐verbenol. Surprisingly, none of these 2nd order Col‐0 individuals emitted measurable amounts of *cis*‐verbenol or verbenone.

From these results, addition of GPPS.SSU does not clearly appear to improve the production of verbenol, nor does it seem to consistently increase α‐pinene emission. Expression of *CYP101*, regardless of which variant, appears to drive production towards *trans‐*verbenol in Arabidopsis Col‐0 plants.

We subsequently assessed the effect of expressing *GPPS.SSU* and *CYP101* variants in the *fps1* V1 line. qRT‐PCR analysis showed the expression of *CYP6DE1*, confirming the presence of the original verbenol pathway construct, and showed expression of *CYP101* variants (*N* = 16) and *GPPS.SSU* (*N* = 14) in the 2nd order transformed lines (Figure 4A). From these 2nd order transformed *fps1 V1* lines, many individuals emitted α‐pinene precursor (*N* = 28) and *trans*‐verbenol (*N* = 15). Only one *fps1* 2nd order transformed line produced *cis*‐verbenol and verbenone (Figure 4C). Interestingly, on average the *fps1* lines that produced α‐pinene seemed to produce 1.54‐fold more of it (regardless of *GPPS.SSU/CYP101* expression) than Col‐0 transformed lines (*p* = 0.0348), with some lines emitting up to 929.3 ng g^−1^ FW h^−1^ α‐pinene. This indicates that limiting FPS1 activity indeed can drive terpenoid production towards GPPS and pinene. There was no clear beneficial effect on verbenol/verbenone production though, but still, it is a useful observation promoting the use of *fps1*. For *fps1* V1 + SSU, three out of seven individuals emitted α‐pinene and only one emitted *trans*‐verbenol. This last individual (*fps1* V1 + SSU 6) showed an emission of 564.3 ng g^−1^ FW h^−1^ α‐pinene and 5.2 ng g^−1^ FW h^−1^ *trans*‐verbenol. For the combination *fps1* V1 + CYP101 FW, three plants out of 12 emitted both α‐pinene and *trans*‐verbenol and one only emitted α‐pinene.

The combination of *fps1* V1 + CYP101 YF had most individuals emitting α‐pinene (8 out of 10) and *trans*‐verbenol (6 out of 10). No relevant emission was detected from the plants carrying only the chloroplast‐targeted variant of CYP101 FW (RecA‐CYP101 FW). However, for the RecA‐CYP101 YF, all individuals showed emission of α‐pinene and five out of 10 showed *trans*‐verbenol. Interestingly, *fps1* V1 + RecA‐CYP101 YF 7 individuals produced both forms of verbenol along with verbenone, while all other lines with any variant of CYP101 only showed *trans*‐verbenol. For this individual, the detected volatile emission was 612.2 ng g^−1^ FW h^−1^ α‐pinene, 11.5 ng g^−1^ FW h^−1^ *cis*‐verbenol, 14.9 ng g^−1^ FW h^−1^ *trans*‐verbenol and 6.2 ng g^−1^ FW h^−1^ verbenone.

When combining GPPS.SSU with any variant of CYP101 in *fps1* background, only three plants out of 33 showed emission of α‐pinene and out of these only one emitted *trans*‐verbenol. This individual (*fps1* V1 + SSU + CYP101 YF 5) emitted 442.4 ng g^−1^ FW h^−1^ α‐pinene and 4.6 ng g^−1^ FW h^−1^ *trans*‐verbenol.

From these results, it can be concluded that overall α‐pinene emission is significantly increased in plants with an *fps1* background. However, CYP101 did not increase the emission of verbenol, and except for one individual it seems to drive the production of verbenol towards *trans*‐verbenol.

### 3.3 *MyrSynt* Expression and Chloroplast Targeting Results in Increased β‐Myrcene Production

From ipsdienol pathway‐transformed plants, there were limited amounts of myrcene (40.3 ng g^−1^ FW h^−1^) produced by introducing *GPPS/MS* from *I. pini* into plants (Figure 2D), likely limiting substrate availability for CYP9T2. To improve myrcene production, we aimed to introduce a dedicated myrcene synthase (MyrSynt) from *Picea abies* (Yin et al. 2017) into the existing Col‐0 I8 line, with or without GPPS.SSU along (Yin et al. 2017).

Relative mRNA expression levels of the 2nd order transgenic plants (offspring from the *MyrSynt* and *GPPS.SSU* super‐transformed Col‐0 I8 line) were measured to evaluate transgene presence (Figure 5A). The expression of *CYP9T2* represents the presence of the ipsdienol pathway construct, while *MyrSynt* and *GPPS.SSU* are the newly transformed genes. Plants showing a high expression of these genes compared to wildtype were further analysed for volatile production via SPME and GC/MS. Individual Col‐0 I8 3 (without *MyrSynt* and *GPPS.SSU* expression) showed emission of β‐myrcene at 27.8 ng g^−1^ FW h^−1^, but not ipsdienol (Figure 2D). However, the addition of GPPS.SSU and MyrSynt resulted in similar limited amounts of β‐myrcene as in the parental line for most individuals (Figure 5D). Emission of β‐myrcene was also scarce since it was only detected in one of three individuals super‐transformed with *SSU*, three out of 11 for *MyrSynt* and only one out of five for the *SSU + MyrSynt* combination.

We also super‐transformed a Camelina (Cam I8) individual with high ipsdienol pathway gene expression (but no detected emission of β‐myrcene nor ipsdienol) with the *GPPS.SSU* and *MyrSynt* constructs (Figure 5C,D). This 2nd order Cam I8 + MyrSynt 1 individual showed a substantial increase in the emission of β‐myrcene (67.8 ng g^−1^ FW h^−1^) compared to the Arabidopsis individuals (up to 40.3 ng g^−1^ FW h^−1^) and Cam I8 (not detectable). As mentioned before, there might be a higher availability of IPP and DMAPP precursors in the chloroplasts. Therefore, we transiently transformed *N. benthamiana* leaves with the ipsdienol pathway genes *GPPS/MS* and *CYP9T2* targeted to the chloroplast (RecA‐I construct) (Figure 5C,D). Compared to WT *N. benthamiana*, we observed a clear β‐myrcene peak in the transformed leaves with an emission of ~270 ng g^−1^ FW h^−1^. In conclusion, co‐expression of *GPPS.SSU* and/or *MyrSynt* did not result in the emission of ipsdienol or increased β‐myrcene production in Arabidopsis. However, in a Camelina background, MyrSynt improved the emission of β‐myrcene. Similarly, chloroplast targeting showed an improvement in β‐myrcene emission after transient transformation in *N. benthamiana* to the highest recorded in this study, while untargeted *GPPS/MS* and *CYP9T2* did not result in detectable β‐myrcene production in *N. benthamiana*.

## 4 Discussion

In this study, we evaluated the potential use of plants as factories for producing bark beetle aggregation pheromones and precursors, which provides an alternative for the expensive chemical synthesis of molecules such as (+)‐*trans*‐verbenol (e.g., 340 € for 25 mg from Toronto Research Chemicals) and ipsdienol (8684 € for 250 mg from Toronto Research Chemicals) This could represent an attractive opportunity since currently the use of pheromone‐baited traps is widespread, but still relies on chemically synthesised molecules (Fettig and Hilszczański 2015).

Another option, in countries where legislation allows the use of genetically modified organisms, could be to use engineered plants as baits or as natural dispensers of pheromones, for example in intercropping setups. These plant‐produced pheromones could then interfere with bark beetle aggregation when trying to locate suitable hosts. Such pheromone‐producing plants could also recruit bark beetle predators that are attracted by bark beetle pheromones when locating their prey (Bakke and Kvamme 1981; Dahlsten et al. 2003; Symonds and Gitau‐Clarke 2016). An advantage of plants as dispensers would be that usually man‐made baits also trap/kill predators from the environment, which has a negative effect on bark beetle population control (Dahlsten et al. 2003).

Our studies (Figures 2 and 5) demonstrate that the *I. pini* enzyme GPPS/MS can produce β‐myrcene, the precursor for ipsdienol, in Arabidopsis and *N. benthamiana*. This confirms in vivo that it is a dual‐function enzyme, which was previously only shown in vitro (Gilg et al. 2005; Martin et al. 2003). As for the final product ipsdienol, no detectable amount was produced in any of our lines. The enzyme converting β‐myrcene into ipsdienol (CYP9T2) has only been characterised in vitro (Sandstrom et al. 2006), so its enzymatic function might be compromised in a plant cell environment. To reduce the possibility that the amount of β‐myrcene would not be sufficient for CYP9T2 to produce a detectable amount of ipsdienol, we decided to super‐transform ipsdienol pathway construct‐carrying lines with a plant‐derived myrcene synthase (MyrSynt) as well as GPPS.SSU, which was shown previously to boost GPP and subsequent terpenoid production (Yin et al. 2017). This transformation was expected to increase the production of the precursor GPP and intermediate β‐myrcene and result in the ipsdienol final product. The result was a similar emission of β‐myrcene (Figure 5D) but still no final product, pointing at CYP9T2 as the most likely limitation for its production. Plant context might influence the proper folding of this enzyme making it non‐functional, or in vivo conditions are far from optimal compared to the in vitro conditions under which it was characterised (Sandstrom et al. 2006). Furthermore, β‐myrcene substrate availability might not be enough for CYP9T2 to produce a detectable amount of ipsdienol. Even though some of our approaches (e.g., MyrSynt in Camelina) show a relatively higher production of β‐myrcene, they do not reach the levels of emission of the α‐pinene intermediate in the verbenol pathway‐transformed individuals.

In an additional effort, we created a version of the ipsdienol pathway construct with both GPPS/MS and CYP9T2 targeted to the chloroplast, where they can take advantage of DMAPP and IPP pools from the MEP pathway. Transient expression in *N. benthamiana* leaves showed an improved emission of β‐myrcene at ~270 ng g^−1^ FW h^−1^, but still no production of ipsdienol (Figure 5D). A possible solution in the future could be the use of CYP9T1, which was also shown to produce ipsdienol in vitro (Sandstrom et al. 2006). Another option could be the use of recently reported candidate genes *Ityp 03153, Ityp 03146*, and *Ityp 01834*, which showed similarity to *CYP9T1/2* in *I. typographus* (Ramakrishnan et al. 2022). A third strategy could be the engineering of a reductase along with *CYP9T2*, as cytochrome P450s require redox partners to perform their oxidation activity (Sadeghi and Gilardi 2013).

The verbenol pathway‐transformed plants have shown not only the *α*‐pinene intermediate, but also the final products *cis‐*verbenol and *trans‐*verbenol, which are both relevant aggregation pheromones for different bark beetle species. The production of α‐pinene was quite high in several transformed plants of different backgrounds, with emission even reaching 2.5 μg g^−1^ FW h^−1^ in the case of Camelina transformed with verbenol pathway genes. Neither transformed Camelina nor Arabidopsis showed obvious growth or development trade‐offs compared to their wild types. At present, there is no evidence that bark beetle pheromone production would lead to excess fitness costs, preventing the use of plants in intercropping or push‐pull approaches. These results could lead to an applied use of plants as a production platform, since α‐pinene itself is also a common constituent of pheromone mixtures for bark beetle trapping, suggesting it can act as an attractant for both bark beetles and their predators (Miller and Asaro 2023; Miller and Rabaglia 2009). In this study, we have shown that the *D. ponderosae* CYP6DE1 enzyme can produce verbenol in a plant background. However, there is variation in pheromone emission from individuals coming from the same line, as well as the cis/trans isomer proportion they emit. This could be explained by growing conditions or other factors not accounted for.

In the case of *trans*‐verbenol, the highest emission was achieved by individual Col‐0 V8 3 with 75.90 ng g^−1^ FW h^−1^, while individual Col‐0 V8 1 achieved the highest *cis*‐verbenol emission with 80.88 ng g^−1^ FW h^−1^. Verbenone, a derivative of these compounds, was also found in these two individuals at 2.69 ng g^−1^ FW h^−1^ and 5.82 ng g^−1^ FW h^−1^, respectively. This suggests that high production of verbenol leads to a partial conversion to verbenone. The compound verbenone is highly important since it is considered an anti‐aggregation bark beetle pheromone (Frühbrodt et al. 2024). This compound is associated with wood decay and may be produced by the symbiotic microorganisms inside bark beetles (Byers et al. 1989; Leufvén et al. 1984). In our plant lines, the production of verbenone is most likely due to autoxidation of verbenol that has accumulated, as previous studies have shown (Bhattacharyya et al. 1960; Dvořáková et al. 2007). A possible application for plants optimised for verbenone production is a push strategy to keep beetles away from economically relevant trees. Also, having plants producing either verbenol or verbenone may allow a push‐pull strategy when these plants are appropriately distributed in a forest (Borden et al. 2006). This could be attained by further optimising the verbenol biosynthetic pathway having a dedicated enzyme for this conversion. However, up to this date, no enzyme is reported either from bark beetles or their associated microorganisms that may have the potential to do it (Frühbrodt et al. 2024).

As pheromone production could still be improved, individuals from the Col‐0 V8 line were super‐transformed with *GPPS.SSU* and/or a variant of *CYP101* (FW/YF with or without chloroplast targeting sequence). Originally a camphor oxidase, CYP101 was shown to produce both *cis‐*verbenol and *trans‐*verbenol in vitro, depending on the amino acid modifications of the enzyme (Bell et al. 2003). This metabolic engineering resulted in individuals expressing the super‐transformed genes at different mRNA levels, but there was no clear correlation between mRNA levels and pheromone emission. Compared to the parental line Col‐0 V8, CYP101 did not increase verbenol emission in the individuals where verbenol was detected. Interestingly, we could observe (Figure 3) that all *CYP101*‐expressing individuals with verbenol production only produced *trans‐*verbenol (*N* = 12), contrasting with their parental line Col‐0 V8, which produced both verbenol isomers. Indeed, with the exception of one individual in the *fps1* V1 background, all plants expressing CYP101 variants produced only *trans‐*verbenol. This suggests that CYP101 may prefer producing *trans‐*verbenol *in planta*, albeit at seemingly low efficiency. This contrasts with previous studies where CYP101 was characterised in vitro and in *E. coli* and produced *cis*‐verbenol in both cases (Bell et al. 2003; Zhou et al. 2024). This difference might occur since the pinene synthase used in this study synthesises (−)‐α‐pinene, while in the above‐mentioned studies they provide (+)‐α‐pinene precursor or a (+)‐α‐pinene synthase. An alternative might be that CYP101 folds differently in plant cells than in bacterial systems and might have different substrate/product preferences. Considering observations in both Col‐0 V8 and *fps1* V1 lines, it appears verbenone is only present when both *cis‐*verbenol and *trans‐*verbenol are detected in the plant (Table S4).

Finally, our results showed that the level of α‐pinene production was significantly higher in *fps1* lines than in Col‐0. This confirms our hypothesis that reduced flow of terpenoids towards FPPS can improve flux towards GPPS. For future GPPS‐dependent terpenoid engineering efforts, a (partial) silencing or inactivation of *FPS* genes by, e.g., CRISPR‐Cas9 could be an important strategy to improve yields.

Production of insect pheromones may also be improved by taking advantage of Camelina and *N. benthamiana* as platforms for pheromone production (Forestier et al. 2021; Löfstedt and Xia 2021; Xia et al. 2021). Before the analyses presented in this study, many transformed individuals in both plant systems (Camelina *N* = 10, *N. benthamiana N* = 64) were analysed via GC/MS using liquid extraction of volatiles with organic solvents such as dichloromethane and heptane, however without success. Even after iterations of different extraction procedures, liquid extraction was not successful in our study to detect bark beetle pheromones. Nonetheless, further improvement of liquid extraction methods, perhaps in combination with enzymatic conversions, could prove useful as bark beetle pheromones and intermediates may be conjugated with other compounds for detoxification. When SPME fibres were used, three out of eight Camelina plants showed α‐pinene emission, while no ipsdienol nor β‐myrcene was detected out of eight individuals (Figure 2D, Table S4). For *N. benthamiana*, two plants were infiltrated with the verbenol pathway construct and one of them showed an emission of 468.1 ng g^−1^ FW h^−1^ α‐pinene, while no β‐myrcene was detected from two individuals infiltrated with the ipsdienol construct (Table S4). However, transient infiltration with chloroplast‐targeted *GPPS/MS* and *CYP9T2* in *N. benthamiana* resulted in ~270 ng g^−1^ FW h^−1^ β‐myrcene emission. Both Camelina and *N. benthamiana* were successfully used to produce the aphid sex pheromone nepetalactone (Ontiveros‐Cisneros et al. 2025), indicating they may still be usable for bark beetle pheromone production given further optimisation. For example, production can be tuned and optimised by targeting the compounds into seeds and to trichomes via specific promoters (Löfstedt and Xia 2021; Wang et al. 2022). Testing different growth regimes, environmental conditions, tissue types and developmental stages may also improve production efficiency.

Further improvement of the bark beetle pheromone‐producing plant lines is needed to sustain commercial production and assess their effects on the behaviour of bark beetles and their predators. Insect studies may also help to determine the best approaches for bark beetle control: plants as biofactories of pheromones for current artificial baits, plant dispensers to interfere with the aggregation of bark beetles, plants as attractants of bark beetle predators, or the use of plants in a push‐pull strategy.

## Author Contributions

O.V.A. and A.O.‐C. conceived the project. O.V.A., A.O.‐C., M.F. and C.L. planned the experiments. A.O.‐C., J.S., S.P., B.D. and H.‐L.W. performed the experiments. O.V.A., A.O.‐C. and J.S. analysed the data. A.O.‐C. and O.V.A. wrote the manuscript with input from the coauthors.

## Funding

This work was supported by the Novo Nordisk Fonden (NNF18OC0034822, NNF23OC0084896). O.V.A. was supported by Vetenskaprådet (2017‐03854, 2021‐04358), Wenner‐Gren Foundation (SSh2023‐0015), Thureus Foundation and Kungliga Fysiografiska Sällskapet i Lund.

## Conflicts of Interest

O.V.A. and A.O.‐C. have registered a patent claim regarding the production of verbenol and verbenone using plants as bio‐factories.

## Supporting information


**Figure S1:** Verbenol and ipsdienol pathway cloning strategy. (A) Synthesised constructs flanked with specific attB sites necessary for MultiSite Gateway cloning. Each gene was codon optimised for Arabidopsis and provided with a 35S promoter and a constitutive terminator. (B) 4‐Fragment Multisite Gateway cloning strategy for verbenol pathway. KanR: kanamycin resistance, ccdB: Toxic protein ccdB, SpecR: spectinomycin resistance, pXZ393b: Destination vector carrying T‐insertion sites for *A. tumefaciens* .
**Figure S2:** Cloning strategy for GPPS.SSU + CYP101. (A) Gibson Assembly procedure to add a RBCS terminator sequence for *GPPS.SSU* and a 35S promoter sequence to *CYP101*. (B) 2‐Fragment MultiSite Gateway cloning following the Gibson Assembly. GA: Gibson Assembly, ccdB: Toxic protein ccdB, KanR: kanamycin resistance, SpecR: spectinomycin resistance, 35S P: 35S promoter, 35S T: 35S terminator, pH2GW7: Destination vector containing a 35S promoter and terminator flanking expression cassette and a hygromycin resistance for positive selection in plant organisms.
**Figure S3:** Transcription analysis and volatile profiling of *N. benthamiana* infiltrated leaves. (A) Relative mRNA expression of verbenol pathway genes (*GPPS, PS* and *CYP6DE1*) and ipsdienol pathway genes (*GPPS/MS* and *CYP9T2*) from infiltrated *N. benthamiana* leaves. WT uninfiltrated plants are used as reference to normalise mRNA expression. (B) α‐pinene (intermediate compound in the verbenol pathway) standard GC/MS retention time. (C) Gas chromatograms of uninfiltrated (WT) and verbenol pathway infiltrated (Ver) *N. benthamiana* leaves. Green shading indicates detection of α‐pinene at the expected 3.8 min retention time.
**Figure S4:** (A) Extracted ions for identification of overlapping α‐pinene and silanol. Ion 75 is characteristic of silanol while 93, 77 and 136 belong to α‐pinene. (B) Mass spectra comparison between a Col‐0 line carrying verbenol pathway (left column) and standard compounds (right column) at the retention time obtained from the standard compounds.
**Table S1:** RT‐qPCR primers used in this project.
**Table S2:** Gibson Assembly primers used in this project.
**Table S3:** Gateway cloning primers used in this project.

**Table S4:** Extended quantification.

## Data Availability

The data that supports the findings of this study is available in the Figures S1–S4, Tables S1–S3, and Table S4 of this article.
