# Supplementary material for: A Plant‐Based Platform for the Production of Bark Beetle Pheromones
Source: Plant Biotechnol J. 2025 Dec 17;24(4):2442–56. doi: 10.1111/pbi.70481 (PMC13140728; doi:10.1111/pbi.70481)
Supplement: Supplementary file 1 — Figure S1: Verbenol and ipsdienol pathway cloning strategy. (A) Synthesised constructs flanked with specific attB sites necessary for MultiSite Gateway cloning. Each gene was codon optimised for Arabidopsis and provided with a 35S promoter and a constitutive terminator. (B) 4‐Fragment Multisite Gateway cloning strategy for verbenol pathway. KanR: kanamycin resistance, ccdB: Toxic protein ccdB, SpecR: spectinomycin resistance, pXZ393b: Destination vector carrying T‐insertion sites for A. tumefaciens . Figure S2: Cloning strategy for GPPS.SSU + CYP101. (A) Gibson Assembly procedure to add a RBCS terminator sequence for GPPS.SSU and a 35S promoter sequence to CYP101. (B) 2‐Fragment MultiSite Gateway cloning following the Gibson Assembly. GA: Gibson Assembly, ccdB: Toxic protein ccdB, KanR: kanamycin resistance, SpecR: spectinomycin resistance, 35S P: 35S promoter, 35S T: 35S terminator, pH2GW7: Destination vector containing a 35S promoter and terminator flanking expression cassette and a hygromycin resistance for positive selection in plant organisms. Figure S3: Transcription analysis and volatile profiling of N. benthamiana infiltrated leaves. (A) Relative mRNA expression of verbenol pathway genes (GPPS, PS and CYP6DE1) and ipsdienol pathway genes (GPPS/MS and CYP9T2) from infiltrated N. benthamiana leaves. WT uninfiltrated plants are used as reference to normalise mRNA expression. (B) α‐pinene (intermediate compound in the verbenol pathway) standard GC/MS retention time. (C) Gas chromatograms of uninfiltrated (WT) and verbenol pathway infiltrated (Ver) N. benthamiana leaves. Green shading indicates detection of α‐pinene at the expected 3.8 min retention time. Figure S4: (A) Extracted ions for identification of overlapping α‐pinene and silanol. Ion 75 is characteristic of silanol while 93, 77 and 136 belong to α‐pinene. (B) Mass spectra comparison between a Col‐0 line carrying verbenol pathway (left column) and standard compounds (right column) at the retention time o [file PBI-24-2442-s002.docx]

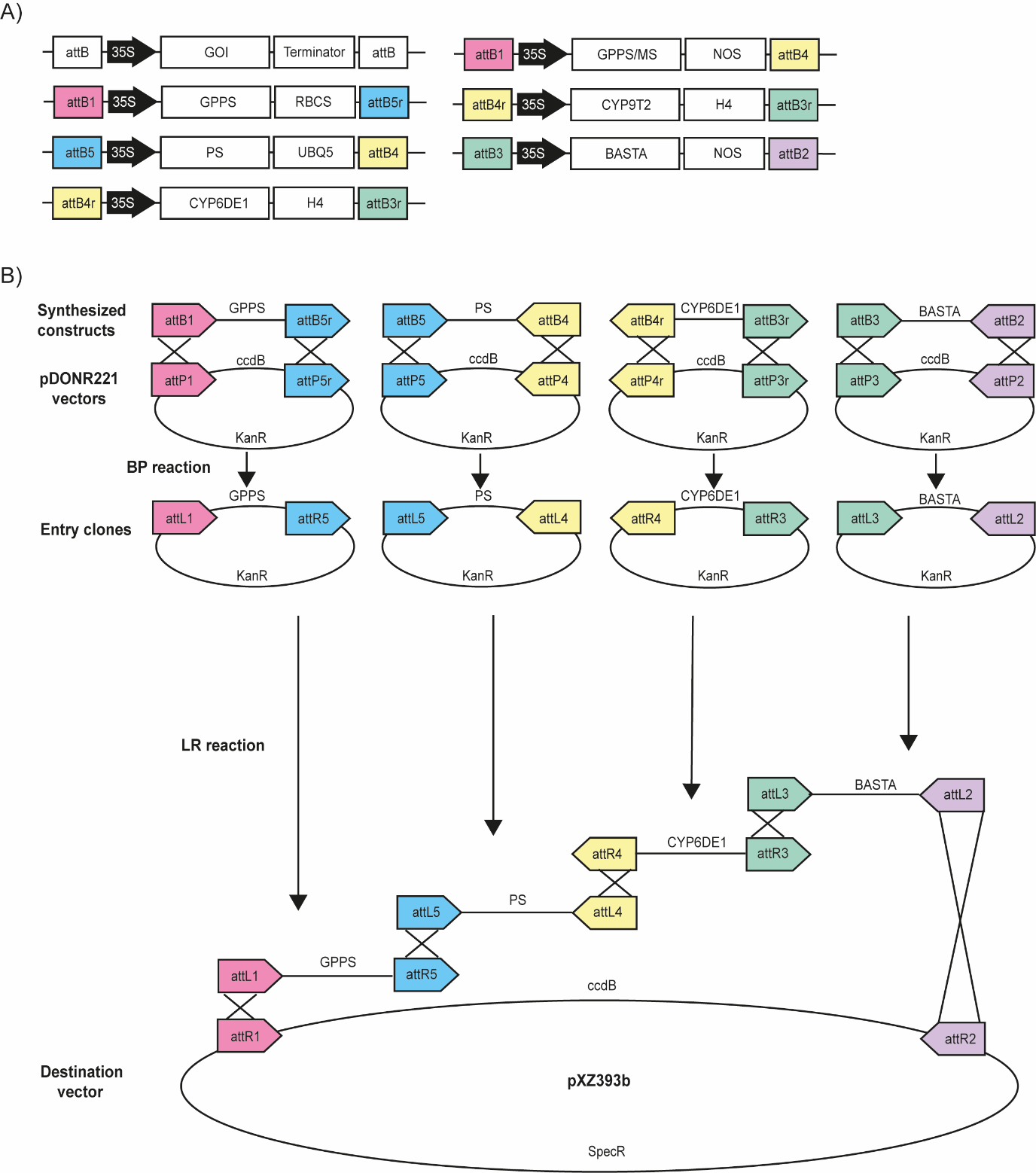


**Figure S1. Verbenol and ipsdienol pathway cloning strategy. A)** Synthesized constructs flanked with specific attB sites necessary for MultiSite Gateway cloning. Each gene was codon optimized for Arabidopsis and provided with a 35S promoter and a constitutive terminator. **B)** 4-fragment Multisite Gateway cloning strategy for verbenol pathway. KanR: kanamycin resistance, ccdB: Toxic protein ccdB, SpecR: spectinomycin resistance, pXZ393b: Destination vector carrying T-insertion sites for *A. tumefaciens*.


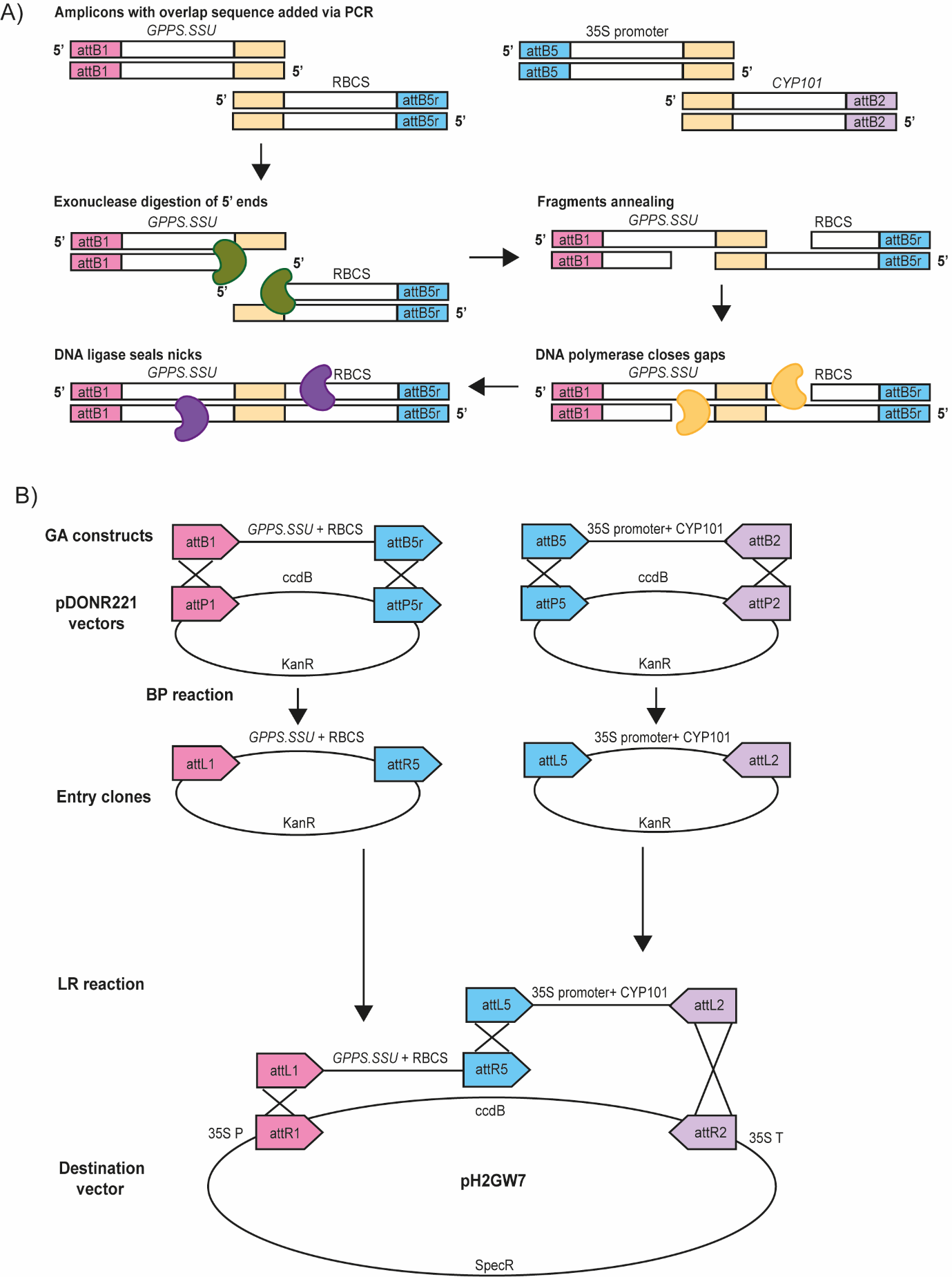


**Figure S2. Cloning strategy for GPPS.SSU + CYP101. A)** Gibson Assembly procedure to add a RBCS terminator sequence for *GPPS.SSU* and a 35S promoter sequence to *CYP101*. B) 2-fragment MultiSite Gateway cloning following the Gibson Assembly. GA: Gibson Assembly, ccdB: Toxic protein ccdB, KanR: kanamycin resistance, SpecR: spectinomycin resistance, 35S P: 35S promoter, 35S T: 35S terminator, pH2GW7: Destination vector containing a 35S promoter and terminator flanking expression cassette and a hygromycin resistance for positive selection in plant organisms.


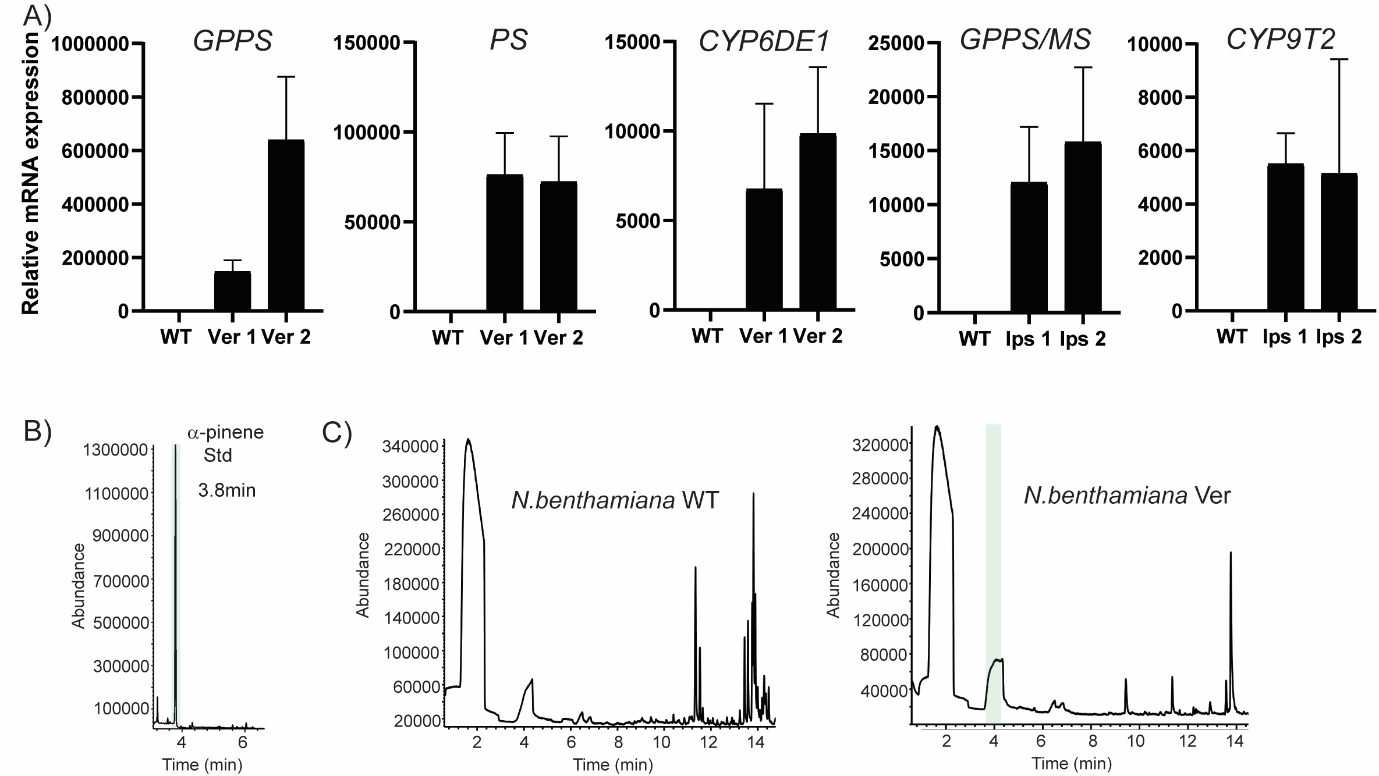


**Figure S3. Transcription analysis and volatile profiling of *N. benthamiana* infiltrated leaves. A)** Relative mRNA expression of verbenol pathway genes *(GPPS, PS* and *CYP6DE1*) and ipsdienol pathway genes (*GPPS/MS* and *CYP9T2*) from infiltrated *N. benthamiana* leaves. WT uninfiltrated plants are used as reference to normalize mRNA expression. **B)** α-pinene (intermediate compound in the verbenol pathway) standard GC/MS retention time. **C)** Gas chromatograms of uninfiltrated (WT) and verbenol pathway infiltrated (Ver) *N. benthamiana* leaves. Green shading indicates detection of α-pinene at the expected 3.8 min retention time.


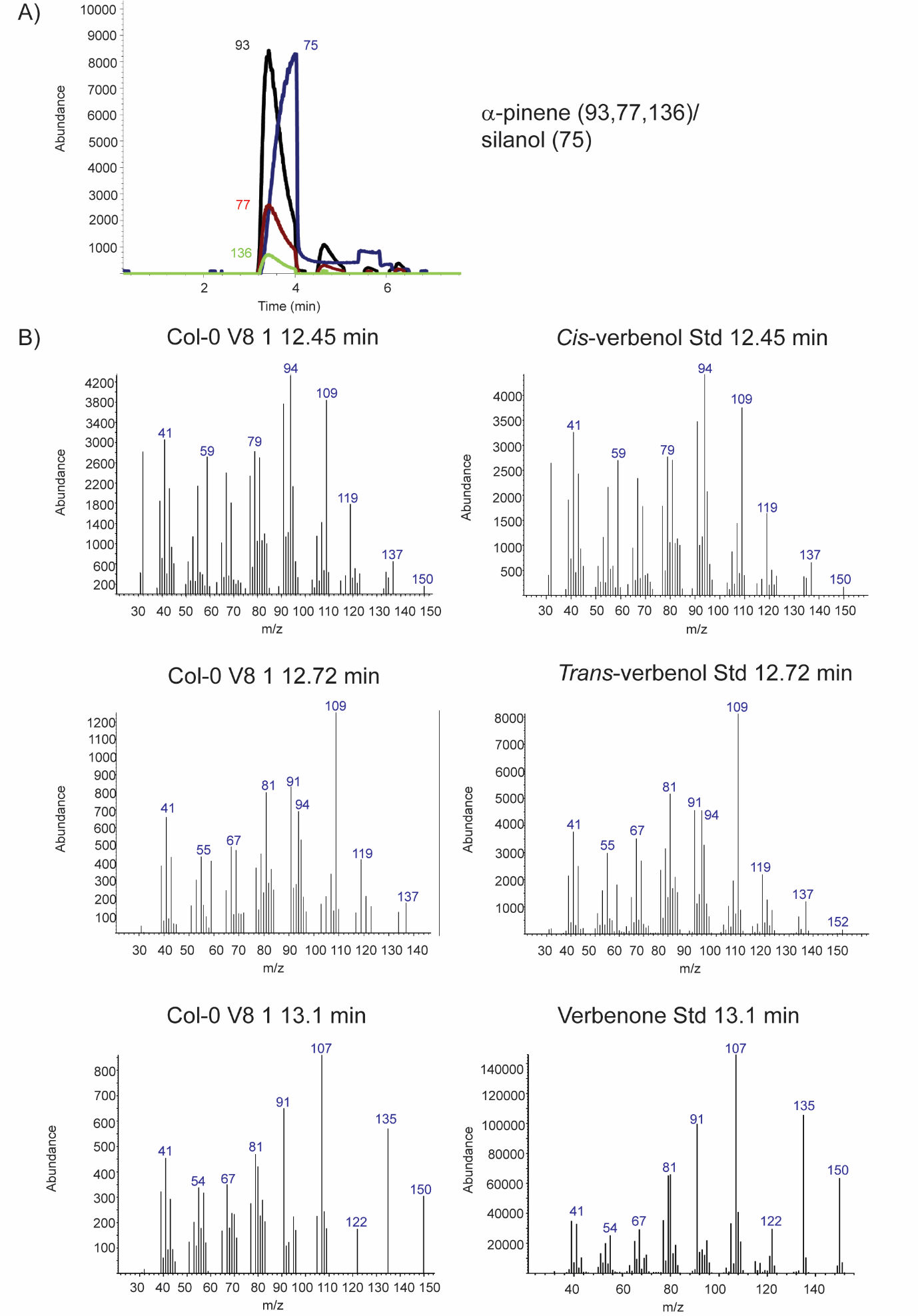
**Figure S4. A)** Extracted ions for identification of overlapping α-pinene and silanol. Ion 75 is characteristic of silanol while 93,77 and 136 belong to α-pinene. **B)** Mass spectra comparison between a Col-0 line carrying verbenol pathway (left column) and standard compounds (right column) at the retention time obtained from the standard compounds.

**Table S1. RT-qPCR primers used in this project.**

| qPCR_PS_F | GGGGAGTCTATCACTCCTCTCG |
| --- | --- |
| qPCR_PS_R | CTGTACACGTGATCGAGGGC |
| qPCR_CYP6DE1_F | ACAGTCTTGGAAAGAGAGGCG |
| qPCR_CYP6DE1_R | GTCAGCAGAGAGTCCAACGA |
| qPCR_GPPS_F | CGCTAGCAGGCAGAACTGAT |
| qPCR_GPPS_R | TCCTCAACTTTTAAGTCGGCAA |
| qPCR_CYP9T2_F | TCTGGGCTGCTAACCTTACTC |
| qPCR_CYP9T2_R | AATTGGCTGCAGCTCTTGTTG |
| qPCR_ACT7Cam_F | AAGAGCAGCTCTTCAGTTGA |
| qPCR_ACT7Cam_R | AACCTCAGGACAACGGAATC |
| qPCR_UBC_F | CTGCGACTCAGGGAATCTTCTA |
| qPCR_UBC_R | TTGTGCCATTGAATTGAACCC |
| qPCR_ACTNB_F | TCCCATTGTGCAATTCATTC |
| qPCR_ACTNB_R | AGCCTTGACCATTCCTGTTC |

**Table S2. Gibson Assembly primers used in this project.**

| GPPS_SSU_R | aaaggggaTCATCATCAGAGGTCCTCTTC |
| --- | --- |
| RBCS_term_F | ctgatgatgaTCCCCTTTCTGGAATATTCAG |
| 35S_prom_MS_R | ggagacatCGTGTCCTCTCCAAATGAAATG |
| MS_F | aggacacgATGTCTCCTGTTTCTGTTGTG |
| 35S_prom_CYP101_RecA_R | tgaatccatCGTGTCCTCTCCAAATGAAATG |
| CYP101_RecA_F | aggacacgATGGATTCACAGCTAGTCTTG |
| 35S_prom_CYP101_R | gtcgtcatCGTGTCCTCTCCAAATGAAATG |
| CYP101_F | aggacacgATGACGACTGAAACCATACAAAG |

**Table S3. Gateway cloning primers used in this project.**

| attB1_C101_WFA_FAL | GGGGACAAGTTTGTACAAAAAAGCAGGCTATGACGACTGAAACCATACAAAGC |
| --- | --- |
| attB2_STOP_MYC | GGGGACCACTTTGTACAAGAAAGCTGGGTTCATCATCACAGATCCTCTTCTG |
| attB1_GPPS_SSU | GGGGACAAGTTTGTACAAAAAAGCAGGCTATGGCCATTAATCTCTCCCATATC |
| attB1_PA_MS | GGGGACAAGTTTGTACAAAAAAGCAGGCTATGTCTCCTGTTTCTGTGGTGC |
